# Biofeedback in dysphonia – progress and challenges^☆^

**DOI:** 10.1016/j.bjorl.2017.07.006

**Published:** 2017-08-19

**Authors:** Geová Oliveira de Amorim, Patrícia Maria Mendes Balata, Laís Guimarães Vieira, Thaís Moura, Hilton Justino da Silva

**Affiliations:** a Universidade Federal de Pernambuco (UFPE), Recife, PE, Brazil; b Hospital dos Servidores do Estado de Pernambuco, Recife, PE, Brazil; c LGV Assessoria e Consultoria Médica, Recife, PE, Brazil

**Keywords:** Speech therapy, Voice, Dysphonia, Electromyography feedback, Fonoaudiologia, Voz, Disfonia, *Feedback* de eletromiografia

## Abstract

**Introduction:**

There is evidence that all the complex machinery involved in speech acts along with the auditory system, and their adjustments can be altered.

**Objective:**

To present the evidence of biofeedback application for treatment of vocal disorders, emphasizing the muscle tension dysphonia.

**Methods:**

A systematic review was conducted in Scielo, Lilacs, PubMed and Web of Sciences databases, using the combination of descriptors, and admitting as inclusion criteria: articles published in journals with editorial committee, reporting cases or experimental or quasi-experimental research on the use of biofeedback in real time as additional source of treatment monitoring of muscle tension dysphonia or for vocal training.

**Results:**

Thirty-three articles were identified in databases, and seven were included in the qualitative synthesis. The beginning of electromyographic biofeedback studies applied to speech therapy were promising and pointed to a new method that enabled good results in muscle tension dysphonia. Nonetheless, the discussion of the results lacked physiological evidence that could serve as their basis. The search for such explanations has become a challenge for speech therapists, and determined two research lines: one dedicated to the improvement of the electromyographic biofeedback methodology for voice disorders, to reduce confounding variables, and the other dedicated to the research of neural processes involved in changing the muscle engram of normal and dysphonic patients.

**Conclusion:**

There is evidence that the electromyographic biofeedback promotes changes in the neural networks responsible for speech, and can change behavior for vocal emissions with quality.

## Resumo

**Introdução:**

Há evidências de que o complexo aparato envolvido no ato da fala, juntamente com o sistema auditivo, e seus ajustes podem ser alterados.

**Objetivo:**

Apresentar evidências da aplicação de *biofeedback* (biorretroalimentação) para tratamento de distúrbios vocais, enfatizando a disfonia de tensão muscular.

**Método:**

Realizou-se uma revisão sistemática nas bases de dados de Scielo, Lilacs, PubMed e Web of Sciences, utilizando a combinação de descritores e admitindo como critérios de inclusão: artigos publicados em revistas com comitê editorial, relatos de casos ou pesquisas experimentais ou quase experimentais sobre o uso de *biofeedback* em tempo real como fonte adicional de monitoração de tratamento de disfonia de tensão muscular ou para treinamento vocal.

**Resultados:**

Trinta e três artigos foram identificados em bases de dados, e sete foram incluídos na síntese qualitativa. O início dos estudos de *biofeedback* eletromiográficos aplicados à terapia fonoaudiológica foram promissores e indicaram um novo método que permitiu bons resultados na disfonia de tensão muscular. No entanto, a discussão dos resultados carecia de evidências fisiológicas que pudessem servir de base. A busca por tais explicações tornou-se um desafio para os fonoaudiólogos e determinou duas linhas de pesquisa: uma dedicada à melhoria da metodologia de *biofeedback* eletromiográfico para distúrbios da voz, para reduzir as variáveis de confusão e outra dedicada à pesquisa de processos neurais envolvidos na alteração do engrama muscular de pacientes normais e disfônicos.

**Conclusão:**

Há evidências de que o biofeedback eletromiográfico promove mudanças nas redes neurais responsáveis pela fala e pode mudar o comportamento para emissões vocais com qualidade.

## Introduction

Oral communication depends on the ability to produce an enormous variety of sounds that together characterize a language. The production of these sounds results in highly specific configurations of the vocal tract, which filters the sound produced in the larynx and modulates it through coordinated movements of the lips, tongue and mandible.^1^ These interactions involve the muscles and the autonomic and central nervous system and are intriguing topic of research involving normal subjects and those with voice disorders.

There is evidence that all the complex machinery involved in speech acts along with the auditory system, which enables the individual to develop natural dynamic adjustments over a speech, in response to perceptible noise pollution, to make the voice more harmonious and adequate to the speech.^2^ These adjustments not always are sufficient to compensate the voice disorder.

Dysphonia constitutes about 40% of the amendments in voice clinics, and the other 60% of the amendments are represented by voice enhancement requests, concerning to deviation from the perceptive properties of the voice that draw attention to the speaker, that means esthetics of voice.^3^ Dysphonia is a communication disorder, represented by any difficulty in vocal emission that prevents the voice from fulfilling its basic role of transmitting verbal and emotional message of an individual,^3^ due to^.^ imbalance of the muscles responsible for voice production.^4^ The altered vocal patterns, as a consequence of dysphonia, result from muscular adjustments, at glottic and supraglottic levels, developed by the subject, in response to the presence or not of laryngeal injury.^5^ In dysphonia by muscle tension, it is assumed that the increased tension of the extrinsic muscles of the larynx elevates the larynx in the neck and disturbs the slope of the laryngeal cartilages.^6^ Consequently, the extrinsic muscles of the larynx are affected and change the tension of the vocal folds, determining the vocal disorder.^7^

The cerebral cortex is the origin of volitional speech control. Nerve impulses are propagated to activate the motor nucleus of the brainstem (ambiguous nucleus, nucleus of the solitary tract and parabrachial core) and the spinal cord, modulating the action of laryngeal muscles, as well as articulators, thoracic and abdominal muscles. The consequences of this activation in voice disorders are new mental representations of the phonatory behavior, which will require a therapeutic process for relearning the function.^8^

The various types of vocal quality are produced by changes in laryngeal kinesiology, whose intrinsic and extrinsic muscles can be in imbalance. This abnormality points out the need to speech diagnosis in order to establish a treatment plan. The non-instrumental diagnostic methods include vocal history, to determine abuse or misuse of the voice; investigation of stress and psychological factors that can alter the vocal production, as well as palpation of the neck, determining muscle tension and elevation of the larynx, with the possibility of quantification with a four to five points Likert scale. These methods, although widely used in clinics and research, have low diagnostic power, being judge-dependents.^7^

The instrumental diagnostic methods include videolaryngoscopy, radiography, needle or surface electromyography and, more recently, although still experimental, functional magnetic resonance imaging.^7^, ^9^, ^10^ Radiography allows to document and assess the increase in anteroposterior muscle contraction, and the presence of nodules, polyps and cysts, but has low diagnostic power for voice disorders.

Electromyography (EMG) enables evaluation of the electrical activity of the laryngeal muscles and therefore of the tension of muscle fibers and the integrity of the laryngeal nervous system.^11^ Additionally, electromyography with electrodes placed on the skin (called surface EMG – sEMG) can be used in operant learning for internal autonomic modification of a behavior through concurrent information to a task. The sEMG allows the individual to observe the physiological process, in real time, contributing to behavioral change, i.e., to establish correction, meeting objectives clearly defined by the physiology of the altered organ function.

The pathophysiology of muscle tension dysphonia is not yet fully elucidated. Some hypothesis admit that the tension in extrinsic muscles move the larynx up in the neck, which alters the slope of laryngeal cartilages. As a result, the intrinsic muscles of the larynx are affected and tense the vocal folds, promoting disphonya.^7^

In the presence of dysphonia, the autonomic and the central nervous system record the sound change and trigger accommodation to reduce the disturbances. This adaptive physiological mechanism can be monitored and viewed through programs that convert the registration of proprioceptive or tactile receptors in images in a process called biofeedback.^12^

Biofeedback occurs during vocal emission, enabling the issuer to make adaptations for correction of vocal disorders. In dysphonia, these corrections can also call for the speaker's attention to the intensity of the disorder, because there is a natural tendency to underestimate it. Thus, in the biofeedback, internal autonomic events, such as muscle tension, are electronically amplified allowing the individual (bio) to receive the information (feedback), which is usually unavailable.^12^

Several studies^13^, ^14^, ^15^, ^16^, ^17^, ^18^ have demonstrated the possibility of biofeedback to help individuals modifying physiological patterns, admitting that an individual, who knows the functioning of his body, can promote oneself changes.

We identified that dysphonic individuals may have difficulty in changing the diverted vocal standard, as a result of the engram of the muscle activity that automated it, whereas mental processes are subject to changes from inputs of visual, auditory and kinesthetic nature, provided by feedback channels.^12^, ^17^, ^19^ These facts rouse our interest in studying dysphonia, in the view of neuroscience.

The objective of this systematic review is to present the evidence of biofeedback application for treatment of vocal disorders, emphasizing the muscle tension dysphonia, as well as for vocal learning of professional singers.

## Methods

Systematic review was conducted in the databases of Scielo, Lilacs, PubMed and Web of Sciences, using the combination of descriptors <feedback>, <voice>, <biofeedback>, <dysphonia>, <tension>, <therapy>, <surface electromyography>, <singers>. Based on information from the abstracts and titles, potentially useful articles were evaluated in the review. It were applied the inclusion criteria, namely: articles published in journals with editorial committee, reporting cases or experimental or quasi-experimental research on the use of biofeedback in real time as additional source of treatment monitoring of muscle tension dysphonia or for vocal training. It was not set any restriction on the study design, language, biofeedback method or method of assessment, due to the scarcity of studies.

Two judges (G.A. and P.M.B.) independently reviewed the titles and abstracts of the located articles and determined those with potential to integrate the review, from which the full texts were obtained for further examination of the judges. At this stage, each of them selected studies that met the defined goals, and the dissenting judgments were resolved by consensus.

While recognizing that the meta-analysis is the gold standard for review, in this article it has not been possible to adopt this methodology given the scarcity of studies and the small number of elements in each study.

## Results

Thirty-three articles were identified in databases, but 16 were excluded already in the analysis of titles and abstracts: four by using biofeedback without surface electromyography; three for being written in German; six systematic reviews; one for showing analysis of the cortex in the voice articulation; and two for having sEMG as the main theme, without biofeedback approach.

**Figure 1:**
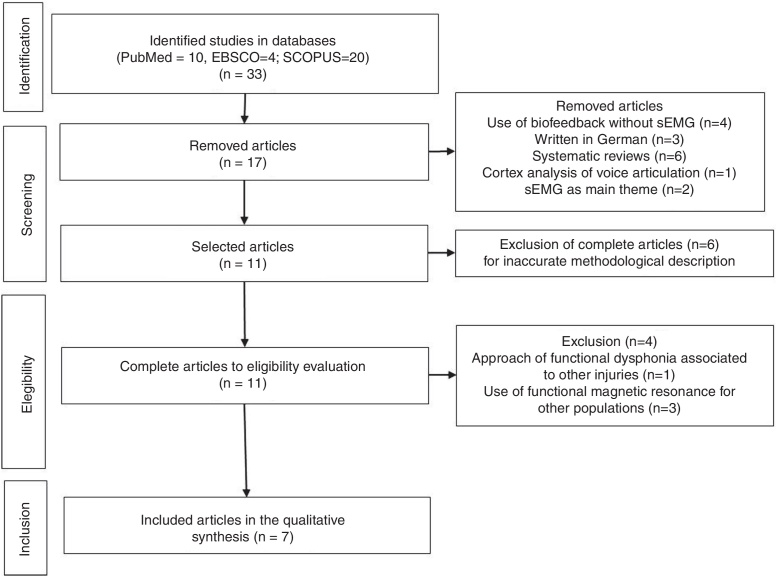
Flowchart of the selection of articles for systematic review.

Seventeen articles were listed by the judges for analysis of the full text, from which resulted the exclusion of six full articles with inaccurate methodological description, leaving eleven articles for evaluation of eligibility. The full reading of the text resulted in the exclusion of four articles, one by approaching functional dysphonia associated with other illness, different from dysphonia, and three by investigating populations without dysphonia using functional magnetic resonance and vocal biofeedback. Seven articles were included in the qualitative synthesis (Fig. 1) (Table 1).

**Table 1:** Description of the studies included in this review.

Authors	Date	Objective	No. of participants (age)	Position of the electrodes in the biofeedback with sEMG	Results
Allen KD, Bernstein B, Chait DH^5^	1991	Reduction in laryngeal muscle tension	1 (8 years)	Ipsilateral and vertically, in parallel alignment on the thyroid membrane with earth-electrode placed on the wrist	Social validity of reduced tension in laryngeal muscles and vocal nodules, with previous surgical indication
Watson TS, Allen SJ, Allen KD^14^	1993	Reduction in muscle tension in vestibular fold dysphonia	1 (26 years)	Ipsilateral and vertically, in parallel alignment along the major axis of the thyroid membrane with earth-electrode placed on the wrist	Disappearance of symptoms and persistence of results in the evaluation, after 6 months
Ruscello^11^	1999	Reduction in phonological errors of acquisition and automation	4 (9, 10, 14, 29 years)	Biofeedback with nasal airflow control Biofeedback self-administered under supervision, having as a standard the correct pronunciation by an instructor Biofeedback self-administered under supervision, with recording of the patient's pronunciation, in usual and correct speech articulation	Social validity of articulatory relearning and reduced laryngeal muscle tension
Warnes E, Allen KD^19^	2005	Reduction in paradoxical vocal fold mobility and respiratory distress	1 (16 years)	Ipsilateral and vertically, in parallel alignment along the major axis of the thyroid membrane, using the thyroid cartilage as additional anatomical marker	Social validity of reduced laryngeal muscle tension, reduced chest pain and crippling respiratory distress, and persistence of results in the evaluation after 6 months
Yiu EM-L, Verdolini K, Chow LPY^9^	2005	Learning of vocal production with laryngeal relaxation Definition of the best conduct in biofeedback (concurrent or terminal)	5 (21–27 years)	Electrodes positioned symmetrically to the midline at four positions: (a) 1 cm from the lip commissure, (b) 0.5 cm from the midline of the chin, (c) 1 cm from the mandibular midline, (d) 0.5 cm from the midline, on the thyroid membrane, and (e) an earth-electrode firmly fixed on the wrist	Absence of biofeedback contribution for vocal learning with relaxation of laryngeal muscles, but presence of orofacial relaxation, attributed to the possible difficulty of normal individuals to reduce laryngeal muscle tension
Kirkpatrick A, McLester JR^15^	2012	Learning of relaxation of the laryngeal depressor muscles for improved voice quality of singers	22 (>21 years)	Bilaterally and before the thyroid cartilage, just above the laryngeal protuberance	Biofeedback helps in activating the depressor muscles of the larynx, increasing the amplitude and tonal quality
Niziolek CA, Guenther FH^2^	2013	Effect of the vocal boundaries in compensatory responses to auditory disturbances in real time	36 (19–33 years)	Visual biofeedback with functional magnetic resonance imaging while hearing the recording of the own normal phonation and with discrete or marked change of a vowel	The acoustic correction of speech errors activates brain areas and may be enhanced by biofeedback

## Discussion

We decided to divide the articles into two groups, one with biofeedback application for correction of muscle tension dysphonia or voice disorders secondary to the laryngeal muscle tension,^12^, ^20^, ^21^, ^22^ and another dedicated to the application of this method in the vocal learning of professional singers.^2^, ^10^, ^16^

The two groups of articles were maintained to contemplate the biofeedback employment opportunities in speech therapy, given that technological advances in imaging studies enabled demonstration of new evidence, either for vocal training, or to treatment of tensional vocal disorders.

### Biofeedback for correction of muscle tension dysphonia or voice disorders secondary to muscle tension

The vocal disorders have been studied for a long time to determine their causes as well as for the definition of effective therapies. Allen, Bernstein and Chait^20^ conducted a study with electromyographic biofeedback on a male child, 8 years old, with hyperfunctional dysphonia not responsive to vocal rehabilitation and relaxation techniques. The patient had vocal nodules and such an intense muscle tension that there was indication for surgery.

The child was subjected to electromyographic evaluations of the larynx muscles tension, and of the voice quality, performed by a professional and the child's parents. The treatment consisted of a 30-minute session twice a week, with visual biofeedback training, followed by 5 min of habituation. At each session, it were held 20 consecutive exercises, first during vocal rest and then during speech, always trying to reduce muscle tension, by observing the feedback of laryngeal muscles. The initial tension reduction criterion was 0.5 μV, in vocal rest, and 5 μV, during speech, in at least 80% of exercises in the three consecutive sessions. The therapy completion criterion was based on the normal baselines for adults, as for children there were no normative data. Following three and 6 months, muscle tension at rest decreased from 7.5 μV to 3.2 μV; at speech, from 49.5 μV to 10 μV, value that was maintained after 6 months.^20^

The differential of this study^20^ was the evaluation of voice quality by the child's parents, featuring social validity of the treatment, i.e., utility and meeting of the parents’ expectations for the results obtained with treatment, giving a value with social applicability.^23^ The social validity requires comparing two basic strategies. The first is the objective evaluation based on normative data. The second is subjective, based on the perception of an expert or of people accustomed to the event under evaluation.^23^ The child's parents, in this experiment, pointed to the validity of the treatment because of the generalization of the effects on the voice, in the residence and other non-clinical settings, indicating stability of the reduction of vocal tension. Another important finding of this research was the evidence of a drastic reduction of vocal nodules, dispensing the surgical treatment previously scheduled.

The second study of this group^22^ describes the case of a 26 year-old male with a history of tension dysphonia after severe laryngitis and viral influenza, with subsequent functional dysphonia of the vestibular vocal fold, 6 months ago. In otorhinolaryngologic consultation, it was identified that the patient made use of analgesic and anti-hypertensive medication that did not promote or affect dysphonia.

Treatment was established with electromyographic biofeedback consisting of two weekly sessions, lasting 20–30 min, with a 10 min habituation, in a room with sound attenuation, 1–20 repeated counts and casual conversation, always in this order, separated by 3 min habituation. Throughout the procedure, the patient was instructed to reduce the tension of the larynx muscles, whether by watching the integrated electromyographic signal or by deep breathing, a relaxation technique that should be repeated in the household twice a day.^22^

The patient was evaluated after three and 6 months, by perceptual, vocal and visual measurements. There was reduction in EMG average levels: from 59 μV to 7 μV in the non-vocalization; from 51 μV to 9 μV in counting; and, from 70 μV to 12 μV in conversation and these reductions were maintained after 6 months. In perceptual voice analysis by the Vocal Screening Profile Buffalo II Wilson^24^ (ranging from 1 – normal – to 5 – severe deviation), the deviation was rated 4, at the beginning of therapy, passing to 1.5, after treatment.^22^

The authors^22^ made an interesting comment concerning the behavior differentiation in counting biofeedback compared to conversation. Contrary to the initial expectations, the intensity of reduction of muscle tension with electromyography biofeedback regarding the conversation was different from that observed in the counting, leading to the conclusion that the counting and the conversation conform different dynamics. Counting involves responses that do not depend on a listener. The conversation requires a partner, whose vocal behavior mediates subtle and explicit social contingencies, requiring changes in the discriminative stimulus for vocal settings. From this, there was indication of starting the treatment with conversation, since it involves more complex tasks and more effort, thus enabling earlier change and achieving results in a shortest period of time.

The third study^12^ was not dedicated to muscle tension dysphonia. Its inclusion derived from the relevance of the approaches presented by the author on explaining the use of biofeedback for phonemic speech disturbances. He tests and proves the effectiveness of the visual electromyographic biofeedback employment to correct phonological disorders in children, refractory to other treatments. The disturbances consisted in the wrong articulation of a phoneme (acquisition) or the misuse of a phoneme already acquired, in a spontaneous context (automation). These changes made the children reading, writing and literacy difficult, from which derived the importance of speech therapy.

In the contextualization to explain the favorable results of electromyographic biofeedback, the author^12^ argues that the primary difference from other speech therapeutic techniques is to reduce the auditory stimulus and increase the visual pathway through instrumental signal. This visualization makes easier for the patient to identify an acquisition or an automation error. The therapist offers the patient the correct phono-articulatory production that enables the recording of the mental model. Then he stimulates emissions by the patient, asking him to mentally attach the task and try to correctly repeat the emission, by using visual feedback.

There were two information sources on feedback in these experiments. One consisted in detecting nasal air flow through pneumotachograph and presenting it on the computer screen as a graphic signal. The second source of information was the spectrogram of the acoustic signal presented in real time so the patient could make his adjustments in order to achieve the target speech. The vision of the alteration was a warning of error production and enabled the individual to modify his performance.

In the first session,^12^ a phonoaudiologist demonstrated the use of equipment and exemplified the right and wrong phonation, recording them to serve as a model to be achieved by the patient. Once assured the patient's understanding about the therapy, the patient performed the exercises, in sessions lasting 60 min, which were monitored by the speech therapist in videoconferencing.

To explain the reasons why adolescent and adult patients persisted with acquisition and automation errors, the author^12^ contextualizes that the phonological development occurs until the age of eight, when the individual reaches the upper limit of speech and language development. From this completeness, the changes will only be possible with therapy, because the individual does not have the necessary information to make the corrections. Other strategies need to be provided so that the individual can form a new engram, well differentiated with respect to the erroneous one, to be recognized and corrected whenever articulated.^12^, ^13^

Ruscello^12^ concludes the paper stating that the biofeedback gives good results not because it is restricted only to the spectrogram visualization, but because the method introduces in the complex of systems involved in speech a new element that has receptive and expressive features, facilitating its integration into the correct speech articulation.

The fourth study^21^ aimed at presenting a case of a female teenager, 16 years old, Caucasian, with history of paradoxical movement of vocal folds signed by video laryngoscopy, associated with chest pain, muscle tension dysphonia, difficulty in breathing and a feeling of suffocation. Despite being treated with breathing exercises for 12 months, progress was not noticed, and a treatment with electromyographic biofeedback was indicated.

The treatment consisted of a weekly session, for 10 weeks. It started with two sessions for electromyographic evaluation of muscle tension, without biofeedback. From then on, each session started with 5 min of vocal basal rest, followed by 10 min of relaxation of muscle tension under visualization of the biofeedback spectrogram, with the guidance of achieving the criterion of reduction of muscle contraction, but without any other guidance. The patient should get the muscle relaxation by trial and error, observing the spectrogram.

Associated with biofeedback, the patient evaluated her perceived adaptation by using a six points Likert scale, and the intensity of chest pain by a 10 points visual analog scale. Additionally, the patient's mother evaluated the pain interference with activities of her daughter, also employing a six points Likert scale, with the objective of determining the social validity of the treatment. Gradually new tension levels were established, lower than the patient's baseline, and the therapy ceased when normal levels have been achieved.

The initial level to be reached was fixed to 10 μV, therefore 2 μV lower than the baseline levels of the patient. New targets were set following the criteria: (a) whenever, in three consecutive sessions, the patient reached the goal or exceeded it achieving greater relaxation, the goal was reduced by 2 μV; (b) if in two subsequent sessions the patient did not reach the set target, the target was increased by 1 μV. In the range of 5 min between one series and another, the patient could see the spectrogram to understand its operation.

After 4 weeks, the patient had not needed to miss any school activity, and did not report chest pain, while her mother reported no interference of vocal disorder in the daily activities of the girl. Despite reporting good results, the authors^21^ considered that the changes may have resulted from nonspecific effects derived from the exercise intensity, more than any biofeedback feature.

The set of articles from that first block took place from 1991 to 2005 period, therefore predating the magnetic resonance imaging studies for the application of voice biofeedback. This set was presented in this systematic review to point out the predominant reasoning within these two decades, founding the hypothesis that this method had the potential to modify the vocal engram. However, the evidences were based on small samples, on case studies with different methodologies, thus without the possibility of generalization, as well emphasized Warnes and Allen.^21^

The mechanisms with which it was sought to explain the effects of electromyographic biofeedback for voice were based on behavioral reinforcement, increased self-confidence and motivational boost, but the results were conflicting.^10^, ^25^, ^26^ Yet the line of research has not been abandoned, although there is scarcity of publications.

This hypothesis fragility motivated studies with professional singers, since the absence of large vocal disorders could facilitate the identification of new evidences on the voice biofeedback. The choice of this population was a form of standardizing some intervening variables. In addition, these studies could clarify gaps on the mechanisms responsible for the results of research and guide more objectively the use of the method in voice therapy.

### Biofeedback in the vocal learning of professionals

From a population with vocal health and good or excellent emission quality, studies have sought to identify the possibility of the electromyographic biofeedback facilitating the biomechanical learning in voice production.

A study aimed to identify a method for facilitation in the acquisition of new skills to voice production with laryngeal relaxation, as well as to determine whether the terminal or concurrent biofeedback optimizes this learning.^10^

After performing a pilot project to determine the type and position of electrodes, as well as the form of fixation, 18 women and four men with a mean age of 22.41 years old (range 19–27 years), with no history of hearing or speech problems and with no experience of vocal therapy, which self-declared healthy, were included in the study. After affixing a pair of electrodes 0.5 cm apart from each other in the midline of the thyroid membrane, and a second pair 1 cm away from the lip commissure, bilaterally, each participant sat in front of two computer screens. In one of these, it were designed 11 blocks of stimulus sentences, to be read aloud looking to relax the larynx by observing the sEMG of the thyroid site, which was designed on the other screen.^10^

After the first two reading stimuli, participants were randomized to receive concurrent or terminal biofeedback in each reading. In the concurrent stimulus, participants watched the muscular electrical activity as they read the sentences. In the terminal biofeedback, they read the sentence and then watched the static graphic of the sEMG. The whole procedure lasted approximately 50–60 min and was repeated after one week, for fixing realx of larynx, although without biofeedback.^10^

The study results did not corroborate other findings. Hence, biofeedback did not appear to facilitate the learning of vocal task. It was observed learning of relaxation in the facial site, but not in the thyroid site, contrary to the initial hypothesis of this study. The third and most interesting finding was the equality in terminal and concurrent biofeedback on learning.

The importance of this experiment was to demonstrate that the concentration of attention on a task (which consisted in reducing muscle tension of normal individuals to values lower than their baseline limit) degrades the motor learning, which pointed to the complexity of vocal learning.

When comparing the results of this study^10^ with these of Warmes and Allen,^21^ it is observed the weakness that marked the evidence on electromyographic biofeedback for voice, until the first decade of the twenty-first century. For patients with muscle tension dysphonia, the results showed that more complex tasks in therapy with electromyographic biofeedback could provide better conditions for good therapeutic results in less time, which was not the case in the experiment with singers. For them, the increased complexity of the experiment caused the non-learning of the voice task for muscle relaxation.

The second study^16^ sought application of the electromyographic biofeedback in the vocal enhancement of singers, to assist them to issue a clear, strong and comfortable voice with a high range, for keeping the low position of the larynx, which protects against injuries in very loud emissions.^16^ Three objectives were set out: to prove the usefulness of sEMG as indicator of the activity of depressor muscles of the larynx; to test the usefulness of the method to teach singers to lower the larynx, activating the depressor muscles, and to maintain this stance as they sing and, ultimately, to determine whether the laryngeal posture improves the quality of the singing tone or promotes a change whatsoever in a scientifically measurable component of the sound spectrum.

Twenty-two trained classical singers were compared to eight untrained singers, using sEMG with electrodes placed bilaterally over the thyroid cartilage in an attempt to isolate the sternohyoid and sternothyroid muscles – primary depressors and stabilizers of the larynx. It was requested for each singer to issue four times the vowel /a/ in a pitch slightly louder than his voice (baritone, tenor, mezzo-soprano and soprano), in order to get out of the vocal comfort zone.

In all cases, the electromyographic biofeedback made it possible to indicate the activity of the depressor muscles of the larynx, as well as facilitated the learning of classical singers to keep low the larynx during singing, to ensure the clearest, most beautiful and harmonic emissions.

Noticing that singers with training were able to improve in up to 12% their baseline performance with a few minutes of training, with no difference between genders, the authors^15^ encouraged to carry out further research with the same design, to accumulate evidence.

Attention must be drawn to the detail of the absence of gender differences in vocal improvement with electromyographic biofeedback, because, according to the authors,^16^ the datum allows to hypothesize that the changes promoted by the therapy should be intense, as they were larger than the anatomical larynx differences in both genders. These changes should also be broad, as they allow relaxation of various laryngeal muscles according to the needs of the patient.

Analyzing the results of research with dysphonic patients and singers, it became evident the need to consider the neural network involved in the issue and the voice setting in a speech, be it emissions of isolated phonemes, speaking or singing.

The comprovation of these hypotheses started from the second decade of the twenty-first century, with the studies on functional magnetic resonance imaging and biofeedback with birds, identifying that hearing changes promoted motor corrections in the singing.^27^ This finding raised the possibility that, in humans, biofeedback could promote change in vocal behavior by restructuring the neural network.

At this time, it was already admitted that the speech motor system depends on the auditory feedback, which is responsible for controlling the vocal articulations from moment to moment. Thus, if during the emission of the voice a disturbance occurs, the auditory system immediately counter-acts and enables corrective adjustments offsetting the imbalance. The explanation for this mechanism is that each sound corresponds to a perceptual auditory area.^28^ These brain regions in the auditory cortex are used to update and refine the motor commands that guide the acoustic signal along these regions.

Similarly to this physiological system, the programs available in the Internet network with auditory or visual feedback enable correction of phonatory acoustic mistakes, but these corrections are still rudimentary. In the nervous system, it is assumed that the auditory perceptions are not created equally, that is, to the sound of the same phoneme, the hearing record can be distinguished, so that the sounds that exceed a certain volume threshold are better identified than others.

This discretionary ability of the nature of the production of a discourse is a hypothesis where it is assumed that small vocal deviations are not perceived by the individual sender, phenomenon called perceptual magnet effect. Not with standing, all deviations that reach a certain threshold are self-recognized and submitted to the vocal adjustment mechanism. The hypothesis was plausible but raised question whether the auditory error depended only on the low level of hearing distance of a limit or intentional target or was triggered by a type of high-level modification.

In 2013, it was held a research^2^ employing electromagnetic biofeedback and functional magnetic resonance imaging (fMRI) to examine the neural networks and determine the changes in response to sudden disturbances of the auditory feedback loop, triggered by unexpected acoustic modifications, admitting that a change of feedback close to a border region might evoke a more intense response than a change lying safely within an acceptable variability of a certain sound in a speech.

Eighteen dexterous subjects, aged between 19 and 33 years old (mean of 23.5 years), with gender distribution of 1:1, took part in a behavioral experiment. All had English as a first language; did not report hearing alterations or speech disorders and had no contraindication to fMRI.

The experiment consisted of two phases, one behavioral and the other imagery. In the behaveoral phase, all were subjected to a pretest to define the parameters of production and perception of speech of different vowels to be employed in the experiment.^2^

In the image capture phase, brain activity was measured by fMRI, during the biofeedback task, within a triggering event of the coordinating mechanism of stimulus. The analysis of fMRI maps demonstrated, by monitoring the auditory feedback, that the speech motor system can quickly correct small articulatory errors. When considering the variability of each subject, one can identify that similar changes can systematically evoke responses of different magnitude, when they occur in different parts of the auditory space. If the changes occur in an area close to the limit for the category in which a sharp change was noticeable, it triggers cortical activity and severe behavioral compensation. If the change was far from the modified category limit, the behavioral change never exceeded 8% of the unchanged acoustic behavior.^2^

This experiment^2^ has shown for the first time that the auditory control with biofeedback is sensitive to linguistic categories and can trigger different behaviors of the neural network, facilitating the automatic corrections of the speech motor system, especially in the superior temporal and inferior frontal regions.

The technological improvement of electromyographs, surface electrodes and computer programs for graphic conversion of electrical current in spectrogram opened new horizons of research in this area, about halfway through the first decade of the twenty-first century. Not with standing, this did not mean greater homogeneity of the therapeutic results in the application of electromyographic biofeedback for voice. Success reports were alternated with the absence of vocal modifications.

The researchers^2^ sought explanations for the disparity of results from two pathways. A group was dedicated to the improvement of the electromyographic biofeedback methodology for voice disorders, seeking to reduce confounding variables, whose activities could explain the inability to form evidence.

A second group was dedicated to the research of neural processes involved in changing the muscle engram of normal and dysphonic patients, resulting in proving the validity of this therapeutic method, a goal that has recently been achieved through study by functional magnetic resonance imaging with singers, whose findings can be generalized to patients with voice disorders.

The directioning of future research should be focused on finding a standardization of biofeedback methods for voice, which is still a difficult challenge. There is evidence as to the validity of the method to guide vocal emissions with quality, but there are no comparative parameters that allow generalizations.

## Conclusions and further research

There is evidence that the electromyographic biofeedback promotes changes in the neural networks responsible for speech, especially superior temporal and inferior parietal cortex, allowing for conditions to promote behavioral change in normal patients or with voice disorder, but this is still a hypothesis, because the discussion of the experiments lacked physiological evidence that could serve as their basis.

## Funding

MCTI/CNPQ/Universal 14/2014 - Faixa A.

## Conflicts of interest

The authors declare no conflicts of interest.
